# Behavioral and Biochemical Impact of Chronic Unpredictable Mild Stress on the Acquisition of Nicotine Conditioned Place Preference in Rats

**DOI:** 10.1007/s12035-017-0585-4

**Published:** 2017-05-08

**Authors:** G. Biala, K. Pekala, A. Boguszewska-Czubara, A. Michalak, M. Kruk-Slomka, K. Grot, B. Budzynska

**Affiliations:** 1 Department of Pharmacology and Pharmacodynamics, Medical University of Lublin, Chodzki 4A Street, 20-093 Lublin, Poland; 2 Department of Medical Chemistry, Medical University of Lublin, Chodzki 4A Street, 20-093 Lublin, Poland

**Keywords:** Chronic mild stress, Nicotine, Conditioned place preference, Oxidative stress, Rats

## Abstract

Addiction is a chronic psychiatric disease which represents a global problem, and stress can increase drug addiction and relapse. Taking into account frequent concomitance of nicotine dependence and stress, the purpose of the present study was to assess behavioral and biochemical effects of chronic unpredictable mild stress (CUMS) exposure on nicotine reward in rats measured in the conditioned place preference (CPP) paradigm. Rats were submitted to the CUMS for 3 weeks and conditioned with nicotine (0.175 mg/kg) for 2 or 3 days. Our results revealed that only CUMS-exposed animals exhibited the CPP after 2 days of conditioning indicating that stressed rats were more sensitive to the rewarding properties of nicotine and that chronic stress exacerbates nicotine preference. Administration of metyrapone (50 mg/kg), a glucocorticosteroid antagonist, and imipramine (15 mg/kg), an antidepressant, abolished nicotine CPP in stressed rats after 2 days of conditioning. The biochemical experiments showed increased markers of oxidative stress after nicotine conditioning for 2 and 3 days, while the CUMS further potentiated pro-oxidative effects of nicotine. Moreover, metyrapone reversed oxidative changes caused by stress and nicotine, while imipramine was not able to overwhelm nicotine- and stress-induced oxidative damages; however, it could exert antioxidant effect if administered repeatedly. The results suggest that recent exposure to a stressor may augment the rewarding effects of nicotine through anhedonia- and stress-related mechanisms. Our study contributes to the understanding of behavioral and biochemical stress-induced modification of the rewarding effects of nicotine on the basis of the development of nicotine dependence.

## Introduction

Drug dependence is a global public health concern with major economic consequences worldwide. While some effective treatments are available for some kind of addictions, high relapse rates still occur. Human and animal studies have identified stress as a critical factor in the drug addiction effects, including acquisition, retention, and relapse to drug abuse [1–8]. For example, clinical studies have demonstrated that exposure to stress or simply the presentation of stress-related cues can induce relapse to drug-seeking behavior in humans [9–11]. Accordingly, exposure to stressors is a factor for relapse to cocaine, and the exaggerated stress response was often observed in former opioids and psychostimulant abusers which have been linked to drug relapse [4, 5, 12]. Stressful situations can also increase alcohol intake in humans [10]. In support of these clinical findings, animal studies report enhanced drug-induced reinstatement of cocaine and amphetamine self-administration and place conditioning or reinforcing properties of cocaine and drug-seeking behavior [13–16]. Enhanced ethanol reward and ethanol intake in the two-bottle choice paradigm after exposure to stressors have also been revealed [6]. Therefore, in animal studies, there has been considerable interest in evaluating potential interactions between exposure to stressors and drug addiction. A great number of studies demonstrate that stress potentiates the rewarding effects of drugs of abuse [12, 17] as already mentioned, but the key mechanisms are still little known.

Concerning nicotine, despite widespread knowledge of the health risks associated with tobacco abuse [18], less than 10% of smokers who attempt to quit each year are successful, and tobacco relapse rates remain high despite current treatments available [19]. The exposure to stress has long been thought to increase the rewarding properties of nicotine and to increase the risk of relapse to nicotine abuse [20, 21], but the mechanisms are not clear. Indeed, exposure to stressors in humans has been shown to increase volume of smoke inhaled, nicotine intake, and desire to smoke [22–24]. It has also been revealed that human subjects commonly report stress as the primary cause for their continued nicotine abuse [25]. In animal models, the effects of stress on nicotine rewarding effects are also demonstrated, as data have shown that an acute stress exposure increases and reinstates nicotine-seeking behavior as measured by reinstatement of nicotine-induced conditioned place preference (CPP) [26] and self-administration [27–30].

Among animal models, the chronic unpredictable mild stress (CUMS) model is the most frequently used and considered one of the most perfect models of depression and stress-related disorders [31, 32]. Its aim is the induction of the state of anhedonia, which is the main symptom of depression in humans, by subjecting animals to the action of mild stress stimuli [32, 33]. In this model, long-term exposure of experimental animals to various mild unpredictable stressors (i.e. restriction, inversion of the light-darkness cycle, deprivation of water or food, wet litter) is related to significant changes in their behavior. All the behavioral changes induced in animals in this model can be reversed by administration of antidepressants. In the context of our experiments, it has to be noted that the CUMS is a widely accepted animal model for inducing anhedonia-related behavior as it mimics the unpredictable intermittent stress exposure and nature of mild stress experience in humans [34, 35]. However, the effects of the CUMS on nicotine rewarding effects in animal models have not been commonly studied so far [36].

Concerning potential mechanisms of above mentioned phenomena, many studies have revealed that various types of stress factors can alter neurotransmitter systems including monoamine (dopamine, serotonin, and noradrenaline), gamma-aminobutyric acid (GABA), and glutamatergic transmission [35, 37, 38]. Both acute stress and the CUMS procedure have been reported to induce changes in neural pathways and neuronal function causing a specific impairment of reward-related behaviors [34, 35, 39]. Specifically, CUMS-induced changes in monoaminergic signaling have all been reported [34, 40–42] implicating important functions of monoamine regulation in the CUMS alterations of mood and reinforcement. Concerning neuroanatomical basis of above mentioned effects taking place following the CUMS, it has been revealed that mice show significant alterations and neuroadaptive changes in neuronal activation of numerous stress-related brain’s regions (i.e. amygdala, hippocampus, and mesocorticolimbic areas) [43] highly associated with chronic drug abuse in general and nicotine in particular [44]. However, little is known on the CUMS effects on regulating drug-mediated conditioned preference behaviors.

Moreover, it is worth mentioning that stress-induced secretion of glucocorticoids seems to have an important role in predisposition to intake of drugs of abuse. The level of corticosterone, an adrenal glucocorticoid, is also increased in response to several types of stressful factors, and the most of the studies confirm that it is the main stress hormone in rodents [45]. Both natural reinforcers and drugs of abuse increase corticosterone secretion. As such, microdialysis studies have shown an increase in the level of corticosterone during an acute stress, especially in the hippocampus, prefrontal cortex, and amygdala [46, 47]. Many literature data have discovered glucocorticoids effects in the mesocorticolimbic system that, in turn, influence motivational and affective behaviors, as well as reinforcing effects of drugs of abuse [48–50]. Accordingly, studies have also demonstrated a positive correlation of stress and drug craving in humans [11], suggesting an activation of reward pathways following exposure to stressor [51]. Thus, treatment aimed at reducing stress could be therapeutically effective for drug addiction.

There are some works indicating the effect of stress on drug-induced CPP [2, 17] as converged lines of evidence indicate that stress increases risk of addictive behaviors and drug-induced reward as already stated [52]. To gain a further understanding of the interaction between the CUMS and nicotine exposure, we studied the effects of concomitant chronic stress exposure on nicotine reward using nicotine-induced CPP after two or three conditioning sessions. The CPP test is one of the most widespread experimental protocols used for measuring drug reward in animals, also in our laboratory [53–56]. We also measured the influence of metyrapone, a glucocorticosteroid inhibitor, and imipramine, a classical antidepressant, on nicotine-induced CPP in order to determine the influence of the hypothalamic-pituitary-adrenal (HPA) axis and anhedonia-related effects on rewarding action of nicotine with and without CUMS in rats.

Recent studies prove participation of reactive oxygen and nitrogen species (ROS and RNS) in pathogenesis and progression of affective disorders including stress-induced depression [57]. Such strong impact of oxidative stress on the central nervous system may arise from the fact that brain is particularly vulnerable to oxidative damages due to high oxygen utilization and therefore high ROS production, poor antioxidant mechanisms in brain, and finally, high concentration of polyunsaturated fatty acids that are substrates for ROS, and compounds easily undergoing redox reactions like metal ions [58–60]. Several researches revealed that depressive disorder is accompanied by oxidant-antioxidant imbalance, which leads to brain damage causing neuroprogression of mood disorders and neurodegeneration [61, 62]. Moreover, nicotine has also been found to increase oxidative stress and decrease antioxidant status [8, 61]. Oxidative changes within fatty acids, proteins, DNA, and mitochondria may damage neurons resulting in cognitive as well as emotional impairments [63, 64]. Normalization of oxidant-antioxidant imbalance was observed after successful pharmacotherapy of affective disorders, and moreover, antioxidant therapy also exhibits antidepressant properties [57].

As the exact amount or concentration of ROS is difficult to measure directly due to their extremely short half-lives, therefore, in indirect way, the markers of oxidative stress are measured. In the series of biochemical experiments, we aimed to determine some important markers as total antioxidant capacity (TAC), activities of antioxidant enzymes: superoxide dismutase (SOD) and glutathione peroxidase (GPx), and the concentration of malondialdehyde (MDA), the main product of lipid peroxidation, in homogenates of isolated brain structures (prefrontal cortex, hippocampus, and cerebellum). The aim of these biochemical experiments was to reveal the influence of the CUMS on the above-mentioned parameters of oxidative stress in rats after 2 or 3 days of saline or nicotine conditioning as well as the influence of metyrapone and imipramine in stressed rats.

In total, both series of our complementary experiments aimed to evaluate behavioral and biochemical impact and complex relationship between nicotine rewarding effects measured in the CPP paradigm in rats and the CUMS, critical for the development and maintenance of nicotine reward and dependence. We have performed these experiments as the continuation of our recent promising data [58] which clearly revealed that CUMS-exposed mice exhibited behavioral alteration like anxiety disorders, the disturbances in memory and depressive effects. Moreover, we have shown that nicotine, after an acute or subchronic administration decreased stress-induced depression- and anxiety-like effect as well as memory deficit in mice also having some pro-oxidative effects.

This kind of experiments would be very important, as the effects of the CUMS on nicotine rewarding effects in animal models and on regulating drug-mediated conditioned preference behaviors have not been commonly studied so far. Taking into consideration frequent concomitance of nicotine abuse and stress which accompanies daily life, finding actual effects of simultaneous exposure to these factors can have great clinical and pharmacological significance.

## Materials and Methods

### Ethics Statement

All experiments were conducted according to the National Institute of Health Guidelines for the Care and Use of Laboratory Animals and to the European Community Council Directive for the Care and Use of Laboratory Animals of 24 November 1986 (86/609/EEC). The protocol was approved by the Committee on the Ethics of Animal Experiments of the Medical University of Lublin (Permit Number: 43/2013). All efforts were made to minimize animal suffering and to reduce the number of animals used.

### Animals

The experiments were carried out on 2-month old naive male Wistar rats, weighing 180–320 g (Farm of Laboratory Animals, Warszawa, Poland) at the beginning of the experiments. The rats were kept under standard laboratory conditions (12 h light/dark cycle, room temperature 21 ± 1 °C) with free access to tap water and laboratory chow (Agropol, Motycz, Poland). Each experimental group consisted of 8–12 rats. All experiments were carried out between 8 a.m. and 7 p.m. The animals were adapted to the laboratory conditions for at least 1 week. They were handled once a day for 7 days preceding the experiments.

### Drugs

The following compounds were tested: nicotine hydrogen tartrate (0.175 mg/kg, Sigma-Aldrich, St. Louis, MO, USA), metyrapone (50 mg/kg, Sigma-Aldrich, St. Louis, MO, USA), and imipramine (15 mg/kg Sigma-Aldrich, St. Louis, MO, USA). Drugs were dissolved in saline solution (0.9% NaCl). Compounds and saline (for control groups) were administered intraperitoneally (i.p.) at a volume of 5 ml/kg. Nicotine dose refers to the base form. The pH of the nicotine solution was adjusted to 7.0. Fresh drug solutions were prepared on each day of behavioral testing. Control groups received saline injections at the same volume and via the same route of administration. The range of doses of drugs was chosen based on literature data, our recently published articles [53–56, 62], and preliminary studies.

### Apparatus

The testing apparatus for the CPP paradigm was already validated in our laboratory [53–56]. To examine the conditioned place preference, eight rectangular apparatus (60 × 35 × 30 cm) made of Plexiglas were used. Each of them was divided into three compartments; two larger rooms with different color and floor structure (25 × 35 cm) were separated by removable guillotine doors from a small gray central area (10 × 10 cm). One room was white with a grooving on the black floor, and another was black with wire mesh on the white floor. Between them, a central neutral compartment with gray walls was located and constituted a “neutral” chamber, which serves as connection and a start compartment. The testing boxes were kept in a soundproof room with neutral masking noise and dim 40-lx illumination. Animal’s behavior was observed on a monitor through a digital video camera system, and the amount of time that the rats spent in each of the two large compartments was recorded using a video tracking software (Karnet, Lublin, Poland).

### Experimental Protocols

Rats subjected to the CUMS procedure (further described in details) were called as stressed rats. Unstressed rats were exposed to behavioral test and not subjected to the CUMS procedure. Nicotine was administered 2 or 3 days during conditioning. Metyrapone was administered 60 min and imipramine 15 min before the CPP test.

At the beginning of the experiments, rats were randomly divided into different groups (8–12 rats in each group). Group I consisted of unstressed saline or nicotine conditioned rats injected with metyrapone or imipramine on the test day, group II consisted of stressed saline or nicotine-conditioned rats injected with metyrapone or imipramine on the test day.

#### CUMS Procedure

The CUMS protocol was performed as described previously [32, 33, 58, 63, 64]. Rats were subjected to different kinds of mild stressors for 21 days preceding the CPP paradigm, which varied from day to day to make the stress procedure unpredictable. These stressors were randomly scheduled repeated throughout the 3 weeks experiment for 2 h daily. There were a total of six stressors: (1) lack of litter, (2) damp sawdust overnight, (3) swimming in cold water for 10 min, (4) tilted cage at 45°, (5) lights on overnight, and (6) food deprivation overnight. Non-stressed rats were left undisturbed in their home cages.

#### CPP

The place conditioning experiment (unbiased design) consisted of pre-conditioning, conditioning, and post-conditioning phases.

1. *Pre-conditioning*

The first phase of the test was used to assess primary place preference and consisted of measuring the time of residence in the two areas for 15 min. During this phase, the animals were placed separately in the central, small gray area with the guillotine doors removed to allow access to the entire apparatus. At this stage was measured the amount of time the rats spent in each of the two large compartments in order to determine the initial preference which was equal in our unbiased experimental design. In the particular experimental setup that we used in our study, the animals did not show a significant preference for either of the compartments during preconditioning phase.

2. *Conditioning*

This stage of the experiment consisted of 30-min sessions, two per day. Guillotine doors, separating the two areas, were closed. Each day consisted of morning and afternoon sessions. Sessions were conducted twice each day with an interval of 4–6 h for 2 or 3 days (days 2–3 or 2–4). One day after pre-conditioning, the rats were randomized and subsequently conditioned as follows. In the morning sessions, the animals were injected with saline and confined in one compartment, whereas on afternoon sessions, the rats received injections of nicotine (0.175 mg/kg, base, i.p.) and were then confined in the second compartment. Injections were administered immediately before confinement in one of the two large compartments, as mentioned above. A dose of 0.175 mg/kg nicotine (base) was chosen for conditioning because it is known to produce reliable CPP in rats after 3 days of conditioning, also under our experimental conditions [53]. The control group received saline every day. The neutral zone was never used during conditioning and was blocked by guillotine doors.

3. *Post-conditioning*

On day 4 or 5, conducted 1 day after the last conditioning trial, animals were placed in the neutral area with the guillotine doors removed and allowed free access to all compartments of apparatus for 15 min. The time spent in the saline- and drug-paired compartments was recorded for each animal. To evaluate the effects of metyrapone and imipramine on the expression of nicotine CPP, on the test day, the rats pretreated with saline or nicotine (as mentioned above) were injected with saline, nicotine (0.175 mg/kg, i.p.), or metyrapone (50 mg/kg–60 min before the test, the dose inactive in the unstressed animals) and imipramine (15 mg/kg–15 min before the test). The time spent by each rat in the two large compartments was recorded during the session lasting 15 min. Comparisons were made to the control group receiving saline injection during the conditioning. The acquisition of the preference was indicated by extended residence time in a space coupled with the injection of nicotine.

#### Locomotor Activity

Locomotor activity was recorded using a photocell apparatus (Porfex, Bialystok, Poland). The rats were placed individually in the Plexiglas boxes (square cages, 60 cm each side) in a sound-attenuated experimental room, under moderate illumination (5 lx). Ambulatory activity (distance traveled) was measured by two rows of the infrared light-sensitive photocells located along the long axis (45 and 100 mm above the floor).

In order to assess the influence of nicotine, metyrapone, or imipramine on the locomotor activity of rats, the animals were placed in Plexiglas boxes directly after the test and then the locomotor activity was measured. Total horizontal activity (distance traveled in meters) was recorded for 15 min.

### Biochemical Procedures

#### Dissection and Homogenization

Immediately after the behavioral assessments, animals were sacrificed by decapitation and the whole brains were removed and rinsed in ice-cold saline to remove blood. Forebrain was dissected out and the cerebrum was discarded. Then, brains were placed on ice, and cortex and hippocampus were separated. The whole brain and the isolated structures of cerebrum, cerebral cortex, and hippocampus were homogenized in 10% Tris buffer (pH 7.4) on ice and centrifuged at 10,000*g* to separate nuclear debris.

#### Measurement of TAC

The total antioxidant capacity of tissue homogenates was determined by ferric-reducing ability of plasma (FRAP) method with modifications for tissue homogenates supernatants. Firstly, standard curve was prepared with FeSO_4_ at concentrations from 0 to 1000 μmol/l. Then, working reagent was prepared by mixing acetate buffer of pH 3.6, 2,4,6-tri-pyridyl-*s*-triazine (10 mmol/l) in 40 mmol/l HCl and aqueous solution of FeCl_3_ (20 mmol/l) in the ratio of 10:1:1. Then, 10 μl of standards and samples were mixed with 20 μl of deionized water and 200 μl of working reagent at 96-well plate. The absorbance was measured at 593 nm after 30 min if incubated at 37 °C. The results were evaluated from the standard curve and recalculated per protein content of supernatants and expressed as micromoles per gram protein.

#### Determination of SOD Activity

Superoxide dismutase activity in prepared homogenates was determined using a commercial available kit RANSOD by Randox. The method employs xantine and xantine oxidase (XOD) to generate superoxide radicals, which react with iodonitrotetrazolium chloride to form red formazan dye. The superoxide dismutase activity is then measured by the degree of inhibition of the reaction. The increase in absorbance at 505 nm is read. The results were recalculated per protein content of supernatants and expressed as unit per gram protein.

#### Determination of GPx Activity

The activity of GPx was measured with the use of ready-to-use diagnostic kits RANSEL by Randox. This method is based on that of Paglia and Valentine [65]. GPx catalyzed the oxidation of glutathione (GSH) by cumene hydroperoxide. In the presence of glutathione reductase (GR) and NADPH, the oxidized glutathione (GSSG) is immediately converted to the reduced form with a concomitant oxidation of NADPH to NADP+. The decrease in absorbance at 340 nm is measured. The results were recalculated per protein content of supernatants and expressed as unit per gram protein.

#### Measurement of Lipid Peroxidation

The level of lipid peroxidation in tissues homogenates was measured by thiobarbituric acid (TBA) test for malondialdehyde (MDA), a low-weight lipid peroxidation by-product. First, the homogenates were deproteinised with 2.8% trichloroacetic acid (TCA), followed by addition of 0.37% TBA in 50 mM NaOH. Then, the samples were heated in a boiling water bath for 20 min to develop a colored MDA–TBA adduct and centrifuged at 10,000*g* for 10 min. The pink chromogen was measured at 532 nm using an Epoch UV–Visible Spectrophotometer against a blank by comparison with the standard curve. A standard was prepared by hydrolysis of 16.4 μl of 1,1,3,3-tetraethoxypropane stock solution in 50 ml of 0.2 mM hydrochloric acid. Such obtained MDA standard (10 mM) was used to prepare standard curve with final concentrations of 1, 2, 3, 5, 7, and 10 μM. The results were evaluated from the standard curve and calculated as micromolar MDA per gram of protein. As the experiment was performed in triplicate, the final results are mean values of them.

#### Determination of Total Protein Content in Tissues Homogenates

The BCA assay (Pierce™ BCA Protein Assay Kit) was employed to measure the protein concentration of each tissue homogenate.

### Statistical Analysis

The data were analyzed by the analysis of variance (ANOVA). For the CPP paradigm, the data are expressed as means ± standard error of mean (SEM) of scores (i.e. the differences between post-conditioning and pre-conditioning time spent in the drug-associated compartment). The statistical analyses were performed using one-way ANOVA with score as variable. Post hoc comparison of means was carried out with the Tukey’s test for multiple comparisons, when appropriate. Locomotor activity was expressed as a number of photocell beam breaks (means ± SEM), and the data were analyzed using repeated measure analysis of variance (ANOVA), as for the biochemical analysis. All statistical tests were performed using GraphPad Prism version 5.01 for Windows (GraphPad Software, USA). The confidence limit of *P* < 0.05 was considered statistically significant.

## Results

All rats showed no significant place preference for the drug-associated compartment before drug conditioning, which indicated that the CPP procedure that we used was of an unbiased design.

### Influence of the CUMS on the Expression of Nicotine-Induced CPP in Rats After 2 days of Conditioning

**Fig. 1:**
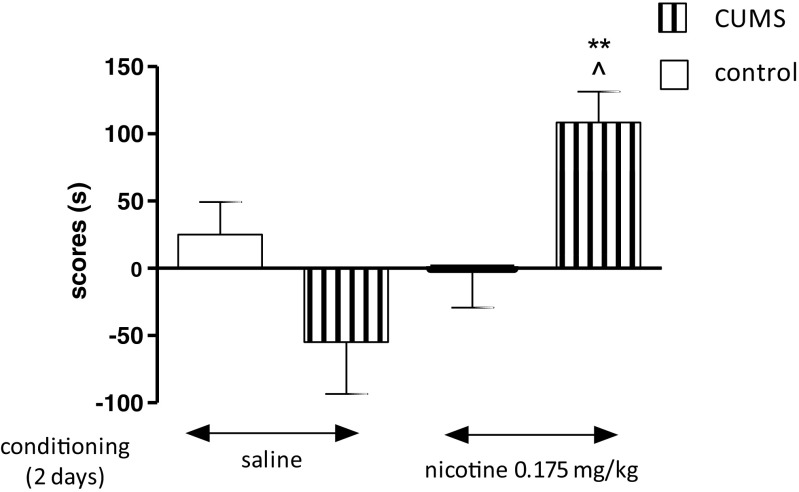
Influence of the CUMS on the expression of nicotine-induced CPP in rats after 2 days of conditioning. Rats were subjected to the CUMS protocol for 21 days. Place preference procedure consisted of pre-conditioning, two conditioning sessions with nicotine (0.175 mg/kg, i.p.), and post-conditioning test. Data represent means ± S.E.M. and are expressed as the difference (in *s*) between post-conditioning and pre-conditioning time spent in the drug-associated compartment. *n* = 8–12 rats per group; **P < 0.01 vs. unstressed nicotine treated rats; ^*P* < 0.05 vs. stressed saline-conditioned rats (Tukey’s test)

Figure 1 shows *the influence of the CUMS on nicotine-induced CPP* [two-way ANOVA: treatment: *F*(1,36) = 5.57, *P* = 0.0238; condition: *F*(1,36) = 10.41, *P* = 0.0027; without treatment × condition interaction: *F*(1,36) = 0.24, *P* = 0.6281]. Administration of nicotine (0.175 mg/kg, two conditioning sessions, days 2–3) did not induce any place preference, shown as no significant values in time spent in the drug-associated compartment during the post-conditioning test phase (day 4, *P* > 0.05, Tukey’s test). Interestingly, post hoc individual comparisons indicated that the exposition to the CUMS protocol significantly increased nicotine-induced CPP after 2 days of conditioning vs. unstressed nicotine-conditioned rats (*P* < 0.01) and stressed saline-conditioned rats (*P* < 0.05) (Fig. 1).

### Influence of Metyrapone on the Expression of Nicotine-Induced CPP in Stressed Rats After 2 days of Conditioning

**Fig. 2:**
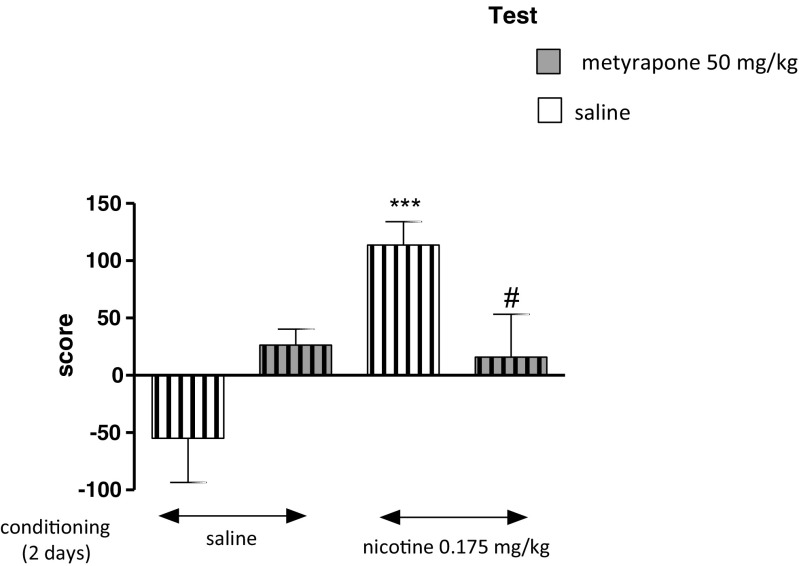
Influence of metyrapone on the expression of nicotine-induced CPP in stressed rats after 2 days of conditioning. Rats were subjected to the CUMS protocol for 21 days. Place preference procedure consisted of pre-conditioning, two conditioning sessions with nicotine (0.175 mg/kg, i.p.) and post-conditioning test. Metyrapone (50 mg/kg) was administered on the test day 60 min before the test. Data represent means ± S.E.M. and are expressed as the difference (in *s*) between post-conditioning and pre-conditioning time spent in the drug-associated compartment. *n* = 8–12 rats per group; ***P < 0.001 vs. stressed saline-conditioned rats; #*P* < 0.05 vs. stressed nicotine-conditioned rats (Tukey’s test)

As shown in Fig. 2, in saline and nicotine 2 days conditioned rats, given saline or metyrapone injection on the test day and exposed to the CUMS protocol, two-way ANOVA analysis revealed that there was a significant effect of pretreatment and treatment [treatment: *F*(1,32) = 1.32, *P* = 0.0027; pretreatment: *F*(1,32) = 45.33, *P* = 0.0071; without treatment × pretreatment interaction: *F*(1,32) = 0.09, *P* = 0.7704]. On the test day, post hoc analysis showed that there were significant differences in scores between nicotine-conditioned and saline-conditioned stressed rats given saline on test day (*P* < 0.001, Tukey’s test). Furthermore, an acute treatment with metyrapone (50 mg/kg) significantly decreased the nicotine-induced CPP in stressed rats as compared with nicotine-conditioned rats given saline injection on the test day (*P* < 0.05) (Fig. 2).

### Influence of Imipramine on the Expression of Nicotine-Induced CPP in Stressed Rats After 2 days of Conditioning

**Fig. 3:**
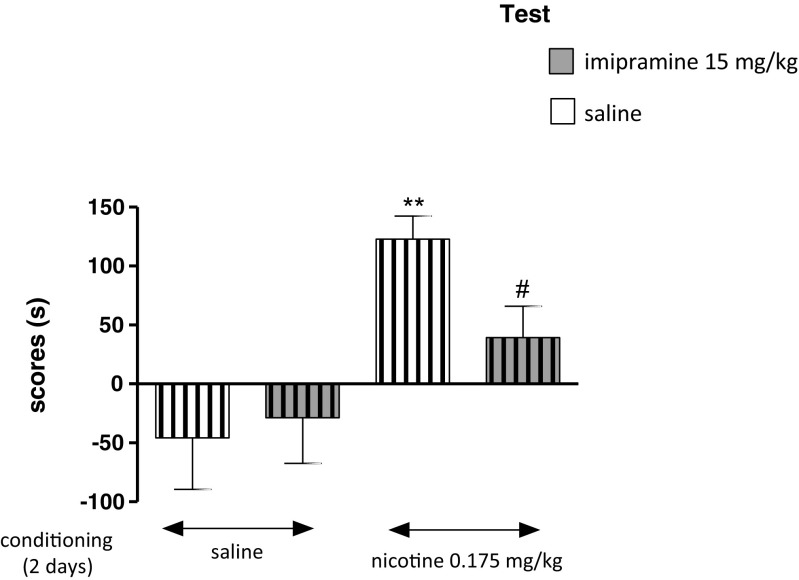
Influence of imipramine on the expression of nicotine-induced CPP in stressed rats after 2 days of conditioning. Rats were subjected to the CUMS protocol for 21 days. Place preference procedure consisted of pre-conditioning, two conditioning sessions with nicotine (0.175 mg/kg, i.p.) and post-conditioning test. Imipramine (15 mg/kg) was administered on the test day 15 min before the test. Data represent means ± S.E.M. and are expressed as the difference (in *s*) between post-conditioning and pre-conditioning time spent in the drug-associated compartment. *n* = 8–12 rats per group; **P < 0.01 vs. stressed saline-conditioned rats; #*P* < 0.05 vs. stressed nicotine-conditioned rats (Tukey’s test)

Figure 3 indicates the effects of imipramine on the expression of nicotine-induced CPP in stressed rats, after 2 days of conditioning [two-way ANOVA: pretreatment: *F*(1,32) = 11.96, *P =* 0.0016, without treatment effect: *F*(1,32) = 2.18, *P* = 0.1500 and treatment × pretreatment interactions: *F*(1,32) = 0.95, *P* = 0.3371]. On the test day, post hoc analysis showed that there were significant differences in scores between nicotine-conditioned and saline-conditioned stressed rats given saline on test day (*P* < 0.01). Furthermore, post hoc Tukey’s test indicated that administration of imipramine (15 mg/kg) to rats exposed to nicotine and the CUMS protocol significantly decreased time spent in the nicotine-associated compartment during the post-conditioning test phase as compared with nicotine-conditioned rats given saline on test day (*P* < 0.05) (Fig. 3).

### Influence of Imipramine on the Expression of Nicotine-Induced CPP in Unstressed Rats after 3 days of Conditioning

**Fig. 4:**
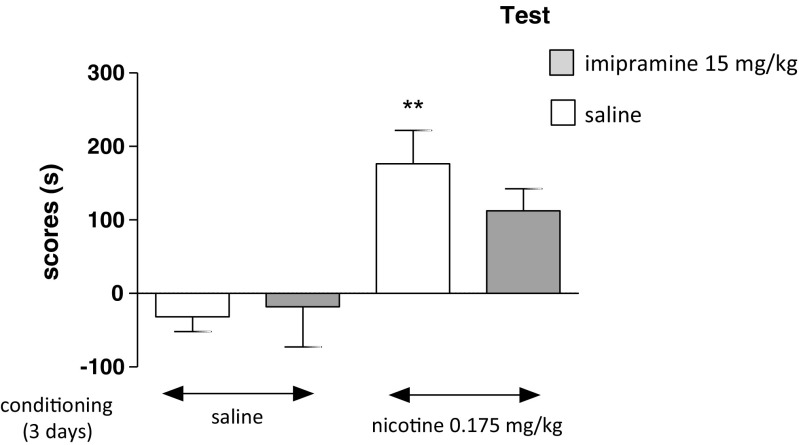
Influence of imipramine on the expression of nicotine-induced CPP in unstressed rats after 3 days of conditioning. Place preference procedure consisted of pre-conditioning, three conditioning sessions with nicotine (0.175 mg/kg, i.p.), and post-conditioning test. Imipramine (15 mg/kg) was administered on the test day 15 min before the test. Data represent means ± S.E.M. and are expressed as the difference (in *s*) between post-conditioning and pre-conditioning time spent in the drug-associated compartment. *n* = 8–12 rats per group; **P < 0.01 vs. saline-conditioned rats (Tukey’s test)

Figure 4 shows the effects of imipramine on the expression of nicotine-induced CPP in unstressed rats [two-way ANOVA: pretreatment: *F*(1,39) = 0.1637, *P* = 0.002 without treatment effect: *F*(1,39) = 1.72, *P* = 0.1968 and treatment × pretreatment interactions: *F*(1,39) = 0.92, *P* = 0.3428]. On the test day, post hoc analysis showed that there were significant differences in scores between nicotine-conditioned and saline-conditioned unstressed rats given saline on test (fifth day) (*P* < 0.01, Tukey’s test) (Fig. 4). Moreover, imipramine did not cause any significant influence on time spent in the nicotine-associated compartment during the post-conditioning test phase in unstressed rats.

### Influence of the CUMS on the Expression of Nicotine-Induced CPP in Rats After 3 days of Conditioning

**Fig. 5:**
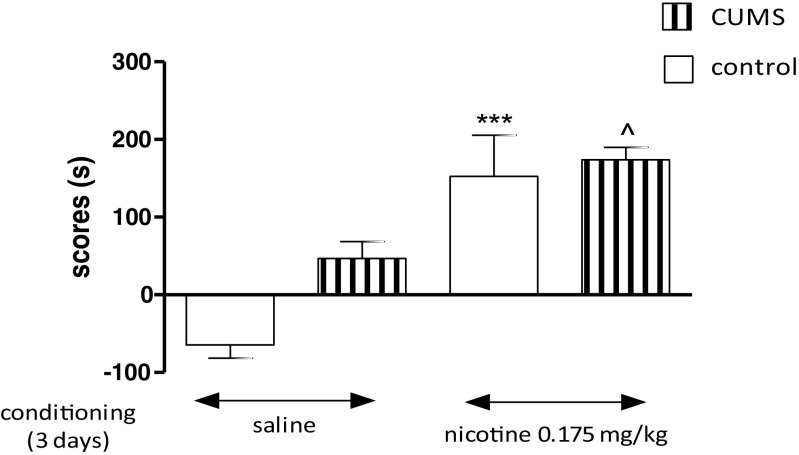
Influence of the CUMS on the expression of nicotine-induced CPP in rats after 3 days of conditioning. Rats were subjected to the CUMS protocol for 21 days. Place preference procedure consisted of pre-conditioning, three conditioning sessions with nicotine (0.175 mg/kg, i.p.), and post-conditioning test. Data represent means ± S.E.M. and are expressed as the difference (in *s*) between post-conditioning and pre-conditioning time spent in the drug-associated compartment. *n* = 8–12 rats per group; ***P < 0.001 vs. unstressed saline-conditioned rats; ^*P* < 0.05 vs. stressed saline-conditioned rats (Tukey’s test)

Figure 5 indicates *the effects of the CUMS on nicotine-induced CPP after 3 days of conditioning* [two-way ANOVA: condition: *F*(1,41) = 58.19, *P* < 0.0001 without treatment effect: *F*(1,41) = 0.13, *P* = 0.7219 and treatment × condition interactions: *F*(1,41) = 0.13, *P* = 0.7219]. Administration of nicotine (0.175 mg/kg, three conditioning sessions, days 2–4) to rats exposed to the CUMS procedure as well as unstressed animals induced place preference, shown as significantly increased values in time spent in the drug-associated compartment during the post-conditioning test phase (day 5) vs. saline-conditioned unstressed and stressed rats (*P* < 0.001 and *P* < 0.05, respectively, post hoc Tukey’s test) (Fig. 5).

### Locomotor Activity

Table 1 indicates the effects of the CUMS in saline- or nicotine-conditioned rats on the horizontal activity measured as the distance traveled by these rats during 15 min. Results showed that the CUMS had significant influence on the observed parameter [two-way ANOVA: treatment: *F*(1,28) = 0.20, *P* = 0.9847, condition: *F*(1,28) = 123.09, *P* < 0.0001, treatment × condition: *F*(1,28) = 0.24, *P* = 0.6315]. Indeed, the CUMS decreased the locomotor activity of the saline- and nicotine-conditioned rats in comparison with unstressed nicotine-conditioned rats (*P* < 0.01) and unstressed saline-conditioned rats (*P* < 0.05).

**Table 1:** Effect of the CUMS on locomotor activity of saline- or nicotine-conditioned rats recorded as distance (m) traveled during 15 min

Procedure	Conditioning	
	Saline	Nicotine
Without CUMS	30.05 ± 3.35	30.53 ± 1.73
After CUMS	19.11 ± 3.46^	18.59 ± 2.79**

Results are expressed as mean ± SEM (*n* = 8)

**P < 0.01 vs. unstressed nicotine-conditioned rats; ^*P* < 0.05 vs. unstressed saline-conditioned rats (Tukey’s test)

Table 2 indicates the influence of imipramine and metyrapone in saline- or nicotine-conditioned rats exposed to the CUMS protocol on the horizontal activity measured as the distance traveled by these rats during 15 min. Our data showed that both drugs had significant influence on the observed parameter [two-way ANOVA: treatment: *F*(1,42) = 26.80, *P* < 0.001, condition: *F*(1,42) = 8.22, *P* = 0.0064, treatment × condition: *F*(1,42) = 11.67, *P* < 0.0001]. Indeed, metyrapone increased the locomotor activity of the stressed rats vs. stressed nicotine-conditioned rats and stressed saline-conditioned rats (*P* < 0.05).

**Table 2:** The influence of imipramine and metyrapone on locomotor activity of saline- or nicotine-conditioned rats exposed to the CUMS protocol, recorded as distance (m) traveled during 15 min

Conditioning	Test		
	Saline	Imipramine	Metyrapone
Saline	19.11 ± 3.46	22.18 ± 3.21	21.03 ± 2.23^
Nicotine	18.59 ± 2.79	23.53 ± 2.42	30.70 ± 3.09*

Results are expressed as mean ± SEM (*n* = 8)

*P < 0.05 vs. stressed nicotine-conditioned rats; ^*P* < 0.05 vs. stressed saline-conditioned rats (Tukey’s test)

#### Biochemical Analysis

Table 3 shows effects of the CUMS on the parameters of oxidative stress (TAC, GPx, SOD, MDA) within the whole brain and its structures (cortex, hippocampus, and cerebellum) in rats after 2 days of saline or nicotine (0.175 mg/kg, i.p.) conditioning. Data are presented for TAC: in the whole brain (two-way ANOVA: condition effect [*F*(1,28) = 12.32, *P* = 0,0015], treatment effect [*F*(1,28) = 38.37, *P* < 0.0001] without interaction [*F*(1,28) = 4.178, *P* = 0.0505]) as well as in single structures as cortex (two-way ANOVA: condition effect [*F*(1,28) = 22.70, *P* < 0.0001], treatment effect [*F*(1,28) = 111.3, *P* < 0.0001] with interaction [*F*(1,28) = 4.744, *P* = 0.0380]), hippocampus (two-way ANOVA: condition effect [*F*(1,28) = 15.49, *P* = 0.0005], treatment effect [*F*(1,28) = 32.59, *P* < 0.0001] without interaction [*F*(1,28) = 2.930, *P* = 0.0980]), and cerebellum (two-way ANOVA: condition effect [*F*(1,28) = 20.30, *P* = 0.0001], treatment effect [*F*(1,28) = 111.2, *P* < 0.0001] without interaction [*F*(1,28) = 4.973, *P* = 0.0339]); GPx: in the whole brain (two-way ANOVA: condition effect [*F*(1,28) = 32.37, *P* < 0.0001], treatment effect [*F*(1,28) = 78.64, *P* < 0.0001] with interaction [*F*(1,28) = 17.62, *P* = 0.0002]) as well as in single structures as cortex (two-way ANOVA: condition effect [*F*(1,28) = 22.14, *P* < 0.0001], treatment effect [*F*(1,28) = 98.90, *P* < 0.0001] without interaction [*F*(1,28) = 0.07969, *P* = 0.7798]), hippocampus (two-way ANOVA: condition effect [*F*(1,28) = 23.87, *P* < 0.0001], treatment effect [*F*(1,28) = 21.84, *P* < 0.0001] without interaction [*F*(1,28) = 1.664, *P* = 0.2076]), and cerebellum (two-way ANOVA: condition effect [*F*(1,28) = 33.56, *P* = 0.0001], treatment effect [*F*(1,28) = 20.97, *P* < 0.0001] without interaction [*F*(1,28) = 0.07451, *P* = 0.7869]); SOD: in the whole brain (two-way ANOVA: condition effect [*F*(1,28) = 10.47, *P* = 0.0031], treatment effect [*F*(1,28) = 10.98, *P* = 0.0026] without interaction [*F*(1,28) = 0.4391, *P* = 0.5130]) as well as in single structures as cortex (two-way ANOVA: condition effect [*F*(1,28) = 22.30, *P* < 0.0001], treatment effect [*F*(1,28) = 22.44, *P* < 0.0001] without interaction [*F*(1,28) = 2.119, *P* = 0.1566]), hippocampus (two-way ANOVA: condition effect [*F*(1,28) = 14.88, *P* < 0.0001], without treatment effect [*F*(1,28) = 3.546, *P* = 0.0701] and interaction [*F*(1,28) = 3.721, *P* = 0.0639]), and cerebellum (two-way ANOVA: treatment effect [*F*(1,28) = 5.827, *P* = 0.0226] without condition effect [*F*(1,28) = 4.111, *P* = 0.0422] and without interaction [*F*(1,28) = 0.06302, *P* = 0.8036]); MDA: in the whole brain (two-way ANOVA: condition effect [*F*(1,28) = 197.3, *P* < 0.0001], treatment effect [*F*(1,28) = 104.9, *P* < 0.0001] with interaction [*F*(1,28) = 9.769, *P* = 0.0041]) as well as in single structures as cortex (two-way ANOVA: condition effect [*F*(1,28) = 317.4, *P* < 0.0001], treatment effect [*F*(1,28) = 423.6, *P* < 0.0001] with interaction [*F*(1,28) = 137.3, *P* < 0.0001]), hippocampus (two-way ANOVA: condition effect [*F*(1,28) = 103.3, *P* < 0.0001], treatment effect [*F*(1,28) = 400.9, *P* < 0.0001] without interaction [*F*(1,28) = 1.113, *P* = 0.3006]), and cerebellum (two-way ANOVA: condition effect [*F*(1,28) = 74.16, *P* < 0.0001], treatment effect [*F*(1,28) = 79.54, *P* < 0.0001] without interaction [*F*(1,28) = 2.934, *P* = 0.0978]).

**Table 3:** Influence of the CUMS on the parameters of oxidative stress (TAC, GPx, SOD, MDA) within the whole brain and its structures (cortex, hippocampus, and cerebellum) in rats after 2 days of saline or nicotine (0.175 mg/kg, i.p.) conditioning

Oxidative stress parameter	Group	Control		CUMS	
	Conditioning	Saline	Nicotine	Saline	Nicotine
TAC	Brain	468.10 ± 3.12	432.50 ± 30.17**	415.30 ± 11.00***	405.90 ± 16.54#
	Cortex	446.00 ± 8.97	425.60 ± 10.22***	408.60 ± 7.892***	401.00 ± 5.40###
	Hippocampus	446.70 ± 21.16	414.20 ± 6.61**	404.00 ± 6.61***	391.20 ± 14.58#
	Cerebellum	449.60 ± 15.52	427.40 ± 6.21***	421.70 ± 9.37***	400.00 ± 7.42###
GPx	Brain	87.96 ± 8.07	83.81 ± 5.99	124.30 ± 8.08***	96.80 ± 9.01#, ^^^
	Cortex	98.33 ± 8.26	84.46 ± 6.68**	125.20 ± 6.59***	112.90 ± 9.58###, ^
	Hippocampus	114.90 ± 5.81	100.30 ± 7.64***	122.90 ± 6.69	114.40 ± 6.48##
	Cerebellum	121.70 ± 3.12	112.60 ± 3.81**	129.70 ± 4.94**	119.70 ± 6.19#, ^^
SOD	Brain	5.69 ± 0.44	5.19 ± 0.48*	5.18 ± 0.25*	4.85 ± 0.20
	Cortex	11.96 ± 1.46	9.87 ± 0.93***	9.87 ± 0.35***	8.76 ± 0.74
	Hippocampus	24.01 ± 3.95	17.71 ± 2.10**	19.86 ± 3.54	17.76 ± 2.32
	Cerebellum	8.64 ± 1.16	8.18 ± 0.59	8.08 ± 0.54	7.49 ± 0.40
MDA	Brain	19.33 ± 1.94	30.07 ± 2.35***	27.68 ± 1.59***	34.52 ± 0.84###, ^^^
	Cortex	28.90 ± 2.85	35.58 ± 2.22***	38.61 ± 2.21***	70.97 ± 4.52###, ^^^
	Hippocampus	22.16 ± 1.51	28.33 ± 1.55***	33.75 ± 1.56***	38.76 ± 1.60###, ^^^
	Cerebellum	17.12 ± 4.26	22.96 ± 1.21***, ###	23.22 ± 1.38***	31.96 ± 1.19###, ^^^

Rats were subjected to the CUMS procedure for 21 days. Data represent means ± SEM, *n* = 10 rats per group

*P < 0.05, **P < 0.01, ***P < 0.001 vs. unstressed saline-conditioned rats; #*P* < 0.05, ##*P* < 0.01, ###*P* < 0.001 vs. unstressed nicotine-conditioned rats; ^*P* < 0.05, ^^*P* < 0.01, ^^^*P* < 0.001 vs. stressed saline-conditioned rats (Tukey’s test)

A post hoc analysis showed that the exposition to the CUMS protocol in nicotine-conditioning rats decreased the values of TAC in the brain (*P* < 0.05), cortex (*P* < 0.001), hippocampus (*P* < 0.05), and cerebellum (*P* < 0.001), increased the values of GPx in the brain (*P* < 0.05), cortex (*P* < 0.001), hippocampus (*P* < 0.01), and cerebellum (*P* < 0.05), increased the values of MDA in the brain, cortex, hippocampus, and cerebellum (*P* < 0.001) but did not influence SOD values as compared with unstressed, nicotine-conditioned animals. Moreover, a post hoc analysis showed that the exposition to the CUMS protocol and saline conditioning decreased the values of TAC in the brain, cortex, hippocampus, and cerebellum (*P* < 0.001), increased the values of GPx in the brain (*P* < 0.001), cortex (*P* < 0.001), and cerebellum (*P* < 0.01), but not in hippocampus, decreased the values of SOD in the brain (*P* < 0.05) and cortex (*P* < 0.001), increased the values of MDA in the brain, cortex, hippocampus, and cerebellum (*P* < 0.001) as compared with unstressed, saline-conditioned animals. Additionally, a post hoc Tukey’s analysis showed that the exposition to the CUMS protocol in nicotine-conditioned rats decreased the values of GPx in the brain (*P* < 0.001), cortex (*P* < 0.05), and cerebellum (*P* < 0.01) and increased the values of MDA in the brain, cortex, hippocampus, and cerebellum (*P* < 0.001) but did not influence TAC and SOD values as compared with stressed, saline-conditioned animals.

Moreover, a post hoc Tukey’s analysis showed that 2 days on nicotine-conditioning in unstressed rats decreased the values of TAC in the brain (*P* < 0.01), cortex (*P* < 0.001), hippocampus (*P* < 0.01), and cerebellum (*P* < 0.001), decreased the values of GPx in cortex (*P* < 0.01), hippocampus (*P* < 0.001), and cerebellum (*P* < 0.01), decreased the values of SOD in the brain (*P* < 0.05), cortex (*P* < 0.001), and hippocampus (*P* < 0.01), increased the values of MDA in the brain, cortex, hippocampus, and cerebellum (*P* < 0.001) as compared with unstressed, saline-treated animals.

Table 4 presents effects of metyrapone (50 mg/kg) on the parameters of oxidative stress (TAC, GPx, SOD, MDA) within the whole brain and its structures (cortex, hippocampus, and cerebellum) in rats after 2 days of saline or nicotine (0.175 mg/kg, i.p.) conditioning. Data are presented for TAC: in the whole brain (two-way ANOVA: condition effect [*F*(1,28) = 5.514, *P* = 0.0262], treatment effect [*F*(1,28) = 6.558, *P* = 0.0161] without interaction [*F*(1,28) = 0.5537, *P* = 0.4630]) as well as in single structures as cortex (two-way ANOVA: conditioning effect [*F*(1,28) = 5.400, *P* = 0.0276], treatment effect [*F*(1,28) = 29.66, *P* < 0.0001] with interaction [*F*(1,28) = 7.220, *P* = 0.0120]), hippocampus (two-way ANOVA: treatment effect [*F*(1,28) = 4.223, *P* = 0.0493] without conditioning effect [*F*(1,28) = 0.3926, *P* = 0.5360] and interaction [*F*(1,28) = 1.329, *P* = 0.2587]), and cerebellum (two-way ANOVA: conditioning effect [*F*(1,28) = 9.377, *P* = 0.0048], treatment effect [*F*(1,28) = 19.62, *P* = 0.0001] without interaction [*F*(1,28) = 4.087, *P* = 0.0529]); GPx: in the whole brain (two-way ANOVA: treatment effect [*F*(1,28) = 82.84, *P* < 0.0001] without conditioning effect [*F*(1,28) = 3.451, *P* = 0.0737] and interaction [*F*(1,28) = 1.488, *P* = 0.2327]) as well as in single structures as cortex (two-way ANOVA: conditioning effect [*F*(1,28) = 5.354, *P* = 0.0282], treatment effect [*F*(1,28) = 9.746, *P* = 0.0041] without interaction [*F*(1,28) = 1.948, *P* = 0.1738]), hippocampus (two-way ANOVA: conditioning effect [*F*(1,28) = 21.73, *P* < 0.0001], treatment effect [*F*(1,28) = 28.80, *P* < 0.0001] with interaction [*F*(1,28) = 6.986, *P* = 0.0133]), and cerebellum (two-way ANOVA: conditioning effect [*F*(1,28) = 35.87, *P* < 0.0001], treatment effect [*F*(1,28) = 42.00, *P* < 0.0001] with interaction [*F*(1,28) = 7.299, *P* = 0.0116]); SOD: in the whole brain (two-way ANOVA: treatment effect [*F*(1,28) = 15.82, *P* = 0.0004] without conditioning effect [*F*(1,28) = 1.323, *P* = 0.2598] and interaction [*F*(1,28) = 0.3551, *P* = 0.560]) as well as in single structures as cortex (two-way ANOVA: conditioning effect [*F*(1,28) = 11.67, *P* = 0.0020], treatment effect [*F*(1,28) = 20.51, *P* = 0.0001] without interaction [*F*(1,28) = 0.07566, *P* = 0.7853]), hippocampus (two-way ANOVA: conditioning effect [*F*(1,28) = 5.211, *P* = 0.0302], treatment effect [*F*(1,28) = 10.29, *P* = 0.0033] without interaction [*F*(1,28) = 0.4690, *P* = 0.4991]), and cerebellum (two-way ANOVA: treatment effect [*F*(1,28) = 16.07, *P* = 0.0004] without conditioning effect [*F*(1,28) = 3.076, *P* = 0.0904] and interaction [*F*(1,28) = 0.3081, *P* = 0.5833]); MDA: in the whole brain (two-way ANOVA: conditioning effect [*F*(1,28) = 57.93, *P* < 0.0001], treatment effect [*F*(1,28) = 21.31, *P* < 0.0001] without interaction [*F*(1,28) = 1.629, *P* = 0.2123]) as well as in single structures as cortex (two-way ANOVA: conditioning effect [*F*(1,28) = 78.05, *P* < 0.0001], treatment effect [*F*(1,28) = 508.7, *P* < 0.0001] with interaction [*F*(1,28) = 22.08, *P* < 0.0001]), hippocampus (two-way ANOVA: conditioning effect [*F*(1,28) = 61.07, *P* < 0.0001], treatment effect [*F*(1,28) = 58.57, *P* < 0.0001] without interaction [*F*(1,28) = 0.2052, *P* = 0.6540]), and cerebellum (two-way ANOVA: conditioning effect [*F*(1,28) = 74.69, *P* < 0.0001], treatment effect [*F*(1, 28) = 178.7, *P* < 0.0001] with interaction [*F*(1,28) = 27.42, *P* < 0.0001]).

**Table 4:** Influence of metyrapone (50 mg/kg) on the parameters of oxidative stress (TAC, GPx, SOD, MDA) within the whole brain and its structures (cortex, hippocampus, and cerebellum) in stressed rats after 2 days of saline or nicotine (0.175 mg/kg, i.p.) conditioning

Oxidative stress parameter	Conditioning	Saline		Nicotine	
	Treatment	Saline	Metyrapone	Saline	Metyrapone
TAC	Brain	415.30 ± 11.00	431.30 ± 18.25	405.90 ± 16.54	414.20 ± 11.36
	Cortex	408.60 ± 7.89	422.40 ± 9.08**	401.00 ± 5.39	400.00 ± 8.30###
	Hippocampus	404.00 ± 18.75	401.90 ± 11.50	391.20 ± 14.58	398.30 ± 11.00
	Cerebellum	421.70 ± 9.37	425.20 ± 11.13	400.00 ± 7.42***	417.10 ± 9.76^^
GPx	Brain	124.30 ± 8.08	126.00 ± 7.28	96.80 ± 9.01***	105.00 ± 7.56###
	Cortex	125.20 ± 6.59	127.70 ± 6.89	112.90 ± 9.58*	123.00 ± 7.35
	Hippocampus	122.90 ± 6.69	145.70 ± 12.78***	114.40 ± 6.48	120.70 ± 6.69###
	Cerebellum	129.70 ± 4.94	152.70 ± 11.02***	119.70 ± 6.19	128.40 ± 6.32###
SOD	Brain	5.18 ± 0.25	5.22 ± 0.11	4.85 ± 0.20*	4.97 ± 0.22
	Cortex	9.87 ± 0.35	10.59 ± 0.94	8.76 ± 0.74*	9.61 ± 0.39#
	Hippocampus	19.86 ± 3.54	22.33 ± 1.17	17.76 ± 2.32	19.09 ± 1.70#
	Cerebellum	8.08 ± 0.54	8.47 ± 0.53	7.49 ± 0.40	7.70 ± 0.43#
MDA	Brain	27.68 ± 1.59	24.95 ± 1.26***	34.52 ± 0.84***	30.69 ± 1.06###, ^^^
	Cortex	38.61 ± 2.21	33.70 ± 2.90*	70.97 ± 4.52***	54.90 ± 3.38###, ^^^
	Hippocampus	33.75 ± 1.56	29.20 ± 2.00***	38.76 ± 1.60***	33.65 ± 1.80###, ^^^
	Cerebellum	23.22 ± 1.38	21.62 ± 1.17	31.96 ± 1.19***	25.44 ± 1.55###, ^^^

Rats were subjected to the CUMS procedure for 21 days. Data represent means ± SEM, *n* = 10 rats per group

*P < 0.05, **P < 0.01, ***P < 0.001 vs. stressed saline-treated, saline- conditioned rats; #*P* < 0.05, ##*P* < 0.01, ###*P* < 0.001 vs. stressed metyrapone-treated, saline-conditioned rats; ^*P* < 0.05, ^^*P* < 0.01, ^^^*P* < 0.001 vs. stressed saline-treated, nicotine-conditioned rats (Tukey’s test)

A post hoc analysis showed that metyrapone administered to saline-conditioned rats subjected to the CMUS procedure increased the values of TAC in cortex (*P* < 0.01), increased the values of GPx in hippocampus (*P* < 0.001) and cerebellum (*P* < 0.001), and decreased the values of MDA in the brain (*P* < 0.001), cortex (*P* < 0.05), and hippocampus (*P* < 0.001) but did not influence SOD values as compared with stressed, saline-conditioned animals. Moreover, a post hoc Tukey’s analysis showed that the exposition to the CUMS protocol in nicotine-conditioned rats decreased the values of TAC in cerebellum (*P* < 0.001), decreased the values of GPx in the brain (*P* < 0.001) and cortex (*P* < 0.05), decreased the values of SOD in the brain (*P* < 0.05) and cortex (*P* < 0.05), and increased the values of MDA in the brain, cortex, hippocampus, and cerebellum (*P* < 0.001) as compared with stressed, saline-conditioned animals.

Moreover, metyrapone administration to stressed nicotine-conditioned rats decreased values of TAC in cortex (*P* < 0.001), decreased the values of GPx in the brain, hippocampus, and cerebellum (*P* < 0.001), decreased the values of SOD in the cortex, hippocampus, and cerebellum (*P* < 0.05), and increased the values of MDA in the brain, cortex, hippocampus, and cerebellum (*P* < 0.001) as compared with metyrapone-injected, saline-conditioned animals. Moreover, a post hoc Tukey’s analysis showed that metyrapone administered to nicotine-conditioned rats subjected to the CMUS procedure increased the values of TAC in the cerebellum (*P* < 0.01) and decreased the values of MDA in the brain, cortex, hippocampus, and cerebellum (*P* < 0.001) but did not influence SOD and GPx values as compared with stressed, saline-treated and nicotine-conditioned animals.

Table 5 presents effects of imipramine (15 mg/kg) on the parameters of oxidative stress (TAC, GPx, SOD, MDA) within the whole brain and its structures (cortex, hippocampus, and cerebellum) in rats after 2 days of saline or nicotine (0.175 mg/kg, i.p.) conditioning. Data are presented for TAC: in the whole brain (two-way ANOVA: treatment effect [*F*(1,28) = 6.699, *P* = 0.0151] without conditioning effect [*F*(1,28) = 0.6140, *P* = 0.4399] and interaction [*F*(1,28) = 0.1269, *P* = 0.7244]) as well as in single structures as cortex (two-way ANOVA: treatment effect [*F*(1,28) = 8.948, *P* = 0.0057] without conditioning effect [*F*(1,28) = 0.007649, *P* = 0.9309] and interaction [*F*(1,28) = 0.1105, *P* = 0.7421]), hippocampus (two-way ANOVA: treatment effect [*F*(1,28) = 7.731, *P* = 0.0096] without conditioning effect [*F*(1,28) = 1.259, *P* = 0.2714] and interaction [*F*(1,28) = 0.1166, *P* = 0.7353]), and cerebellum (two-way ANOVA: treatment effect [*F*(1,28) = 19.86, *P* = 0.0001] without conditioning effect [*F*(1,28) = 0.01306, *P* = 0.9098] and interaction [*F*(1,28) = 1.110, *P* = 0.3010]); GPx: in the whole brain (two-way ANOVA: treatment effect [*F*(1,28) = 42.18, *P* < 0.0001] and interaction [*F*(1,28) = 5.110, *P* = 0.0318]) without conditioning effect [*F*(1,28) = 2.053, *P* = 0.1630] as well as in single structures as cortex (two-way ANOVA: treatment effect [*F*(1,28) = 14.54, *P* = 0.0007] without conditioning effect [*F*(1,28) = 0.05234, *P* = 0.8207] and interaction [*F*(1,28) = 0.7690, *P* = 0.3880]), hippocampus (two-way ANOVA: conditioning effect [*F*(1,28) = 7.814, *P* = 0.0093], treatment effect [*F*(1,28) = 15.75, *P* = 0.0005] without interaction [*F*(1,28) = 2.722, *P* = 0.1101]), and cerebellum (two-way ANOVA: conditioning effect [*F*(1,28) = 11.98, *P* = 0.0017], treatment effect [*F*(1,28) = 48.84, *P* < 0.0001] with interaction [*F*(1,28) = 7.224, *P* = 0.0120]); SOD: in the whole brain (two-way ANOVA: treatment effect [*F*(1,28) = 17.45, *P* = 0.0003] without conditioning effect [*F*(1,28) = 0.8929, *P* = 0.3528] and interaction [*F*(1,28) = 0.1172, *P* = 0.7346]) as well as in single structures as cortex (two-way ANOVA: treatment effect [*F*(1,28) = 26.77, *P* < 0.0001] without conditioning effect [*F*(1,28) = 3.350, *P* = 0.0779] and interaction [*F*(1,28) = 8.675, *P* = 0.9926]), hippocampus (two-way ANOVA: treatment effect [*F*(1,28) = 9.112, *P* = 0.0054] without conditioning effect [*F*(1,28) = 3.203, *P* = 0.0843] and interaction [*F*(1,28) = 0.2632, *P* = 0.6119]), and cerebellum (two-way ANOVA: treatment effect [*F*(1,28) = 15.36, *P* = 0.0005] without conditioning effect [*F*(1,28) = 0.3181, *P* = 0.5773] and interaction [*F*(1,28) = 0.09667, *P* = 0.7582]); MDA: in the whole brain (two-way ANOVA: treatment effect [*F*(1,28) = 15.9, *P* < 0.0001] without conditioning effect [*F*(1,28) = 2.133, *P* = 0.1553] and interaction [*F*(1,28) = 0.3684, *P* = 0.5488]) as well as in single structures as cortex (two-way ANOVA: treatment effect [*F*(1,28) = 374.6, *P* < 0.0001] without conditioning effect [*F*(1,28) = 3.789, *P* = 0.617] and interaction [*F*(1,28) = 0.07046, *P* = 0.7926]), hippocampus (two-way ANOVA: treatment effect [*F*(1,28) = 32.58, *P* < 0.0001] without conditioning effect [*F*(1,28) = 3.841, *P* = 0.0600] and interaction [*F*(1,28) = 0.8424, *P* = 0.3665]), and cerebellum (two-way ANOVA: conditioning effect [*F*(1,28) = 14.87, *P* = 0.0006], treatment effect [*F*(1,28) = 152.3, *P* < 0.0001] without interaction [*F*(1,28) = 0.8128, *P* = 0.3750]).

**Table 5:** Influence of imipramine (15 mg/kg) on the parameters of oxidative stress (TAC, GPx, SOD, MDA) within the whole brain and its structures (cortex, hippocampus, and cerebellum) in stressed rats after 2 days of saline or nicotine (0.175 mg/kg, i.p.) conditioning

Oxidative stress parameter	Conditioning	Saline		Nicotine	
	Treatment	Saline	Imipramine	Saline	Imipramine
TAC	Brain	415.30 ± 11.00	420.10 ± 6.68	405.90 ± 16.54	407.70 ± 11.33
	Cortex	408.60 ± 7.89	409.80 ± 10.95	401.00 ± 5.398	400.30 ± 7.08
	Hippocampus	404.00 ± 6,61	407.20 ± 10.88	391.20 ± 14.58	397.20 ± 12.78
	Cerebellum	421.70 ± 9.37	418.00 ± 14.39	400.00 ± 7.42**	404.60 ± 12.10
GPx	Brain	124.30 ± 8.08	121.70 ± 6.82	96.80 ± 9.01***	108.49 ± 12.33#
	Cortex	125.20 ± 6.59	123.50 ± 8.11	112.90 ± 9.58*	115.80 ± 4.30
	Hippocampus	122.90 ± 6.69	139.20 ± 16.87*	114.40 ± 6.48	118.60 ± 6.48##
	Cerebellum	129.70 ± 4.94	144.00 ± 9.33***	119.70 ± 6.19*	121.50 ± 4.83###
SOD	Brain	5.18 ± 0.25	5.08 ± 0.15	4.85 ± 0.20*	4.80 ± 0.21**
	Cortex	9.87 ± 0.35	10.26 ± 0.79	8.76 ± 0.74**	9.15 ± 0.43##
	Hippocampus	19.86 ± 3.54	21.79 ± 1.43	17.76 ± 2.32	18.83 ± 1.59
	Cerebellum	8.08 ± 0.54	8.11 ± 0.28	7.49 ± 0.40*	7.61 ± 0.28
MDA	Brain	27.68 ± 1.59	28.77 ± 1.59	34.52 ± 0.84***	34.97 ± 1.77###
	Cortex	38.61 ± 2.21	34.86 ± 7.26	70.97 ± 4.52***	68.12 ± 3.73###
	Hippocampus	33.75 ± 1.56	30.74 ± 1.96	38.76 ± 1.60*	37.67 ± 5.12###
	Cerebellum	23.22 ± 1.38	21.27 ± 1.76	31.96 ± 1.19***	28.82 ± 2.75###, ^

Rats were subjected to the CUMS procedure for 21 days. Data represent means ± SEM, *n* = 10 rats per group

*P < 0.05, **P < 0.01, ***P < 0.001 vs. stressed saline-treated, saline-conditioned rats; #*P* < 0.05, ##*P* < 0.01, ###*P* < 0.001 vs. stressed imipramine-treated, saline-conditioned rats; ^*P* < 0.05, ^^*P* < 0.01, ^^^*P* < 0.001 vs. stressed saline-treated, nicotine-conditioned rats (Tukey’s test)

A post hoc analysis showed that imipramine administered to nicotine-conditioned rats subjected to the CUMS procedure decreased the values of GPx in the brain (*P* < 0.05), hippocampus (*P* < 0.01), and cerebellum (*P* < 0.001), decreased the values of SOD in cortex (*P* < 0.01), and increased the values of MDA in the brain, cortex, hippocampus, and cerebellum (*P* < 0.001) but did not influence TAC values as compared with imipramine-treated, saline-conditioned animals. Moreover, in these rats, imipramine decreased the values of MDA in the cerebellum (*P* < 0.05) as compared with stressed, saline-treated, nicotine-conditioned animals. A post hoc Tukey’s analysis also showed that imipramine administered to saline-conditioned rats subjected to the CUMS procedure increased the values of GPx in hippocampus (*P* < 0.05) and cerebellum (*P* < 0.001) as compared with saline-treated, saline-conditioned animals.

Table 6 presents effects of the CUMS on the parameters of oxidative stress (TAC, GPx, SOD, MDA) within the whole brain and its structures (cortex, hippocampus, and cerebellum) in rats after 3 days of saline or nicotine (0.175 mg/kg, i.p.) conditioning. Data are presented for TAC: in the whole brain (two-way ANOVA: treatment effect [*F*(1,28) = 54.11, *P* < 0.0001], conditioning effect [*F*(1,28) = 55.01, *P* < 0.0001] without interaction [*F*(1,28) = 1.116, *P* = 0.2895]) as well as in single structures as cortex (two-way ANOVA: treatment effect [*F*(1,28) = 266.9, *P* < 0.0001], conditioning effect [*F*(1,28) = 201.4, *P* < 0.0001] and interaction [*F*(1,28) = 24.97, *P* < 0.0001]), hippocampus (two-way ANOVA: treatment effect [*F*(1,28) = 68.55, *P* < 0.0001], conditioning effect [*F*(1,28) = 62.66, *P* < 0.0001] and interaction [*F*(1,28) = 6.059, *P* = 0.0203]), and cerebellum (two-way ANOVA: treatment effect [*F*(1,28) = 33.96, *P* < 0.0001] and conditioning effect [*F*(1,28) = 43.48, *P* < 0.0001] without interaction [*F*(1,28) = 2.897, *P* = 0.0998]); GPx: in the whole brain (two-way ANOVA: treatment effect [*F*(1,28) = 127.1, *P* < 0.0001], conditioning effect [*F*(1,28) = 121.4, *P* < 0.0001]) and interaction [*F*(1,28) = 14.91, *P* = 0.0006] as well as in single structures as cortex (two-way ANOVA: treatment effect [*F*(1,28) = 168.4, *P* < 0.0001] and conditioning effect [*F*(1,28) = 36.78, *P* < 0.0001] without interaction [*F*(1,28) = 0.0033, *P* = 0.9549]), hippocampus (two-way ANOVA: treatment effect [*F*(1,28) = 55.57, *P* < 0.0001], conditioning effect [*F*(1,28) = 20.52, *P* = 0.0001] and interaction [*F*(1,28) = 6.819, *P* = 0.0143]) and cerebellum (two-way ANOVA: treatment effect [*F*(1,28) = 46.10, *P* < 0.0001], and conditioning effect [*F*(1,28) = 48.65, *P* < 0.0001] without interaction [*F*(1,28) = 0.4516, *P* = 0.5071]); SOD: in the whole brain (two-way ANOVA: conditioning effect [*F*(1,28) = 46.78, *P* < 0.0001] without treatment effect [*F*(1,28) = 1.520, *P* = 0.2279] and interaction [*F*(1,28) = 0.2624, *P* = 0.6125]) as well as in single structures as cortex (two-way ANOVA: treatment effect [*F*(1,28) = 5.697, *P* = 0.0240] and conditioning effect [*F*(1,28) = 63.08, *P* < 0.0001] without interaction [*F*(1,28) = 1.941, *P* = 0.1746]), hippocampus (two-way ANOVA: treatment effect [*F*(1,28) = 7.529, *P* = 0.0105], conditioning effect [*F*(1,28) = 75.12, *P* < 0.0001] and interaction [*F*(1,28) = 6.529, *P* = 0.0163]), and cerebellum (two-way ANOVA: treatment effect [*F*(1,28) = 10.56, *P* = 0.0030] and conditioning effect [*F*(1,28) = 23.09, *P* < 0.0001] without interaction [*F*(1,28) = 0.0037, *P* = 0.9517]); MDA: in the whole brain (two-way ANOVA: treatment effect [*F*(1,28) = 171.9, *P* < 0.0001], conditioning effect [*F*(1,28) = 513.7, *P* < 0.0001] and interaction [*F*(1,28) = 69.28, *P* < 0.0001]) as well as in single structures as cortex (two-way ANOVA: treatment effect [*F*(1,28) = 571.4, *P* < 0.0001], conditioning effect [*F*(1,28) = 650.7, *P* < 0.0001] and interaction [*F*(1,28) = 131.9, *P* < 0.0001]), hippocampus (two-way ANOVA: treatment effect [*F*(1,28) = 199.2, *P* < 0.0001], conditioning effect [*F*(1,28) = 206.1, *P* < 0.0001] and interaction [*F*(1,28) = 4.407, *P* = 0.0449]), and cerebellum (two-way ANOVA: treatment effect [*F*(1,28) = 236.1, *P* < 0.0001] and conditioning effect [*F*(1,28) = 424.2, *P* < 0.0001] without interaction [*F*(1,28) = 0.1748, *P* = 0.6790]).

**Table 6:** Influence of the CUMS on the parameters of oxidative stress (TAC, GPx, SOD, MDA) within the whole brain and its structures (cortex, hippocampus, and cerebellum) in rats after 3 days of saline or nicotine (0.175 mg/kg, i.p.) conditioning

Oxidative stress parameter	Group	Control		CUMS	
	Conditioning	Saline	Nicotine	Saline	Nicotine
TAC	Brain	459.60 ± 4.86	428.20 ± 8.30***	428.50 ± 14.66***	402.60 ± 9.83###, ^^^
	Cortex	477.70 ± 7.80	417.80 ± 7.54***	411.10 ± 5.06***	394.90 ± 7.06###, ^^^
	Hippocampus	443.80 ± 9.59	406.70 ± 12.83***	405.40 ± 4.84***	396.20 ± 7.84##, ^^
	Cerebellum	450.90 ± 7.14	414.10 ± 7.77***	417.50 ± 18.34***	397.10 ± 8.18#, ^^
GPx	Brain	93.02 ± 5.07	77.80 ± 6.52***	125.20 ± 5.46***	100.30 ± 9.52###, ^^^
	Cortex	96.76 ± 4.38	82.29 ± 6.52***	127.30 ± 5.93***	117.40 ± 4.97###, ^^
	Hippocampus	112.30 ± 5.40	96.67 ± 8.20***	122.90 ± 6.69*	118.60 ± 7.68###
	Cerebellum	123.30 ± 4.17	106.80 ± 6.95***	136.50 ± 6.11***	125.80 ± 4.73###, ^^^
SOD	Brain	5.51 ± 0.30	4.95 ± 0.29***	5.45 ± 0.24	4.81 ± 0.19^^^
	Cortex	11.87 ± 0.82	9.89 ± 0.93***	11.57 ± 1.00	9.15 ± 0.43^^^
	Hippocampus	25.22 ± 1.55	17.49 ± 2.47***	21.57 ± 0.92**	18.59 ± 0.86^^^
	Cerebellum	9.39 ± 0.30	8.11 ± 0.79*	8.53 ± 0.28	7.74 ± 0.41^^^
MDA	Brain	19.11 ± 1.40	33.09 ± 1.41***	28.78 ± 1.05***	32.67 ± 0.70#, ^^^
	Cortex	21.34 ± 1.01	35.58 ± 2.22***	33.95 ± 1.10***	71.28 ± 2.92###, ^^^
	Hippocampus	22.06 ± 0.54	29.65 ± 0.04***	29.50 ± 2.93***	39.11 ± 1.68###, ^^^
	Cerebellum	13.06 ± 0.56	25.40 ± 1.95***, ###	21.46 ± 1.30***, ###	31.05 ± 2.23***

Rats were subjected to the CUMS procedure for 21 days. Data represent means ± SEM, *n* = 10 rats per group

*P < 0.05, **P < 0.01, ***P < 0.001 vs. unstressed saline-conditioned rats; #*P* < 0.05, ##*P* < 0.01, ###*P* < 0.001 vs. unstressed nicotine-conditioned rats; ^*P* < 0.05, ^^*P* < 0.01, ^^^*p* < 0.001 vs. stressed saline-conditioned rats (Tukey’s test)

A post hoc analysis showed that the exposition to the CUMS protocol in 3 days-nicotine-conditioned rats decreased the values of TAC in the brain (*P* < 0.001), cortex (*P* < 0.001), hippocampus (*P* < 0.01), and cerebellum (*P* < 0.01), decreased the values of GPx in the brain, cortex (*P* < 0.01), and cerebellum (*P* < 0.001), decreased the values of SOD in the brain, cortex, hippocampus, and cerebellum (*P* < 0.001), increased the values of MDA in the brain, cortex, and hippocampus (*P* < 0.001) as compared with stressed, saline-conditioned animals. Moreover, exposition to the CUMS protocol in saline-conditioned rats decreased the values of TAC in the brain, cortex, hippocampus, and cerebellum (*P* < 0.001), increased the values of GPx in the brain (*P* < 0.001), cortex (*P* < 0.001), hippocampus (*P* < 0.05), and cerebellum (*P* < 0.001), decreased the values of SOD in hippocampus (*P* < 0.01), and increased the values of MDA in the brain, cortex, hippocampus, and cerebellum (*P* < 0.001) as compared with unstressed, saline-conditioned animals.

Additionally, nicotine injection decreased the values of TAC in the brain, cortex, hippocampus, and cerebellum (*P* < 0.001), decreased the values of GPx in the brain, cortex, hippocampus, and cerebellum (*P* < 0.001), decreased the values of SOD in the brain (*P* < 0.001), cortex (*P* < 0.001), hippocampus (*P* < 0.001), and cerebellum (*P* < 0.05), and increased the values of MDA in the brain, cortex, hippocampus, and cerebellum (*P* < 0.001) as compared with unstressed, saline-conditioned animals. Finally, a post hoc Tukey’s analysis showed that the exposition to the CUMS protocol in nicotine-conditioned rats decreased TAC in the brain (*P* < 0.001), cortex (*P* < 0.001), hippocampus (*P* < 0.01), and cerebellum (*P* < 0.05), increased the values of GPx in the brain, cortex, hippocampus, and cerebellum (*P* < 0.001), and increased the values of MDA in the brain (*P* < 0.05), cortex (*P* < 0.001), and hippocampus (*P* < 0.001) but did not influence SOD values as compared with unstressed, nicotine-conditioned animals.

Table 7 presents effects of imipramine (15 mg/kg) on the parameters of oxidative stress (TAC, GPx, SOD, MDA) within the whole brain and its structures (cortex, hippocampus, and cerebellum) in rats after 3 days of saline or nicotine (0.175 mg/kg, i.p.) conditioning. Data are presented for TAC: in the whole brain (two-way ANOVA: treatment effect [*F*(1,28) = 34.92, *P* < 0.0001] and interaction [*F*(1,28) = 5.486, *P* = 0.0265] without conditioning effect [*F*(1,28) = 0.6939, *P* = 0.4119]) as well as in single structures as cortex (two-way ANOVA: treatment effect [*F*(1,28) = 49.91, *P* < 0.0001] and interaction [*F*(1,28) = 6.475, *P* = 0.0167] without conditioning effect [*F*(1,28) = 2.691, *P* = 0.1121]), hippocampus (two-way ANOVA: conditioning effect [*F*(1,28) = 4.368, *P* = 0.0458], treatment effect [*F*(1,28) = 30.18, *P* < 0.0001] without interaction [*F*(1,28) = 2.959, *P* = 0.0964]), and cerebellum (two-way ANOVA: treatment effect [*F*(1,28) = 14.25, *P* = 0.0008] without conditioning effect [*F*(1,28) = 0.5117, *P* = 0.4803] and interaction [*F*(1,28) = 1.003, *P* = 0.3252]); GPx: in the whole brain (two-way ANOVA: treatment effect [*F*(1,28) = 93.44, *P* < 0.0001] and interaction [*F*(1,28) = 6.295, *P* = 0.0182] without conditioning effect [*F*(1,28) = 0.0071, *P* = 0.9331]) as well as in single structures as cortex (two-way ANOVA: treatment effect [*F*(1,28) = 14.30, *P* = 0.0008] and interaction [*F*(1,28) = 4.224, *P* = 0.0493] without conditioning effect [*F*(1,28) = 0.08280, *P* = 0.7757]), hippocampus (two-way ANOVA: conditioning effect [*F*(1,28) = 4.731, *P* = 0.0382], treatment effect [*F*(1,28) = 10.88, *P* = 0.0027] with interaction [*F*(1,28) = 4.854, *P* = 0.0360]), and cerebellum (two-way ANOVA: treatment effect [*F*(1,28) = 10.31, *P* < 0.0033] without conditioning effect [*F*(1,28) = 0.6521, *P* = 0.4262] and interaction [*F*(1,28) = 3.598, *P* = 0.0682]); SOD: in the whole brain (two-way ANOVA: treatment effect [*F*(1,28) = 59.07, *P* < 0.0001] without conditioning effect [*F*(1,28) = 2.563, *P* = 0.1206] and interaction [*F*(1,28) = 3.150, *P* = 0.0868]) as well as in single structures as cortex (two-way ANOVA: treatment effect [*F*(1,28) = 77.88, *P* < 0.0001] without conditioning effect [*F*(1,28) = 0.1093, *P* = 0.7434] and interaction [*F*(1,28) = 3.324, *P* = 0.0790]), hippocampus (two-way ANOVA: treatment effect [*F*(1,28) = 28.32, *P* < 0.001] and interaction [*F*(1,28) = 8.677, *P* = 0.0064] without conditioning effect [*F*(1,28) = 0.2811, *P* = 0.6001]), and cerebellum (two-way ANOVA: treatment effect [*F*(1,28) = 16.99, *P* = 0.0003] without conditioning effect [*F*(1,28) = 0.7032, *P* = 0.4088] and interaction [*F*(1,28) = 1.926, *P* = 0.1761]); MDA: in the whole brain (two-way ANOVA: conditioning effect [*F*(1,28) = 6.009, *P* = 0.0207], treatment effect [*F*(1,28) = 84.90, *P* < 0.0001] without interaction [*F*(1,28) = 4.137, *P* = 0.0515]) as well as in single structures as cortex (two-way ANOVA: treatment effect [*F*(1,28) = 539.4, *p* < 0.0001] without conditioning effect [*F*(1,28) = 2.936, *P* = 0.0977] and interaction [*F*(1,28) = 3.511, *P* = 0.0714]), hippocampus (two-way ANOVA: treatment effect [*F*(1,28) = 172.9, *P* < 0.0001] without conditioning effect [*F*(1,28) = 0.1986, *P* < 0.6593] and interaction [*F*(1,28) = 1.603, *P* = 0.2159]), and cerebellum (two-way ANOVA: treatment effect [*F*(1,28) = 178.7, *P* < 0.0001] and conditioning effect [*F*(1,28) = 74.69, *P* < 0.0001] with interaction [*F*(1,28) = 27.42, *P* < 0.0001]).

**Table 7:** Influence of imipramine (15 mg/kg) on the parameters of oxidative stress (TAC, GPx, SOD, MDA) within the whole brain and its structures (cortex, hippocampus, and cerebellum) in stressed rats after 3 days of saline or nicotine (0.175 mg/kg, i.p.) conditioning

Oxidative stress parameter	Conditioning	Saline		Nicotine	
	Treatment	Saline	Imipramine	Saline	Imipramine
TAC	Brain	428.50 ± 14.66	420.80 ± 6.56	386.40 ± 21.87***	402.60 ± 9.83
	Cortex	411.10 ± 5.06	408.40 ± 6.44	382.40 ± 12.98***	394.90 ± 7.06#, ^
	Hippocampus	405.40 ± 4.84	406.40 ± 4.47	385.90 ± 11.36***	396.20 ± 7.84
	Cerebellum	417.50 ± 18.34	409.70 ± 8.67	395.80 ± 13.49*	397.10 ± 8.18
GPx	Brain	125.20 ± 5.46	118.90 ± 7.00	93.56 ± 6.84***	100.30 ± 9.52###
	Cortex	127.30 ± 5.93	121.60 ± 6.90	113.10 ± 9.05**	117.40 ± 4.97
	Hippocampus	122.90 ± 6.69	139.20 ± 16.87*	118.70 ± 3.48	118.60 ± 7.68##
	Cerebellum	136.50 ± 6.11	129.30 ± 11.01	122.90 ± 6.78**	125.80 ± 4.73
SOD	Brain	5.45 ± 0.24	5.22 ± 0.19	4.80 ± 0.14***	4.81 ± 0.19##
	Cortex	11.57 ± 1.00	11.00 ± 0.82	8.75 ± 0.62***	9.15 ± 0.43###
	Hippocampus	21.57 ± 0.92	19.80 ± 0.92	17.36 ± 2.42***	18.59 ± 0.86**
	Cerebellum	8.53 ± 0.28	8.40 ± 0.20	7.21 ± 1.24**	7.74 ± 0.41
MDA	Brain	28.78 ± 1.05	28.54 ± 2.69	35.25 ± 1.20***	32.67 ± 0.70###, ^
	Cortex	33.95 ± 1.10	39.32 ± 6.01	71.52 ± 5.08***	71.28 ± 2.92###
	Hippocampus	29.50 ± 2.93	30.71 ± 1.09	39.69 ± 1.85***	39.11 ± 1.68###
	Cerebellum	21.46 ± 1.30	22.74 ± 1.77	33.27 ± 2.07***	31.05 ± 2.23###

Rats were subjected to the CUMS procedure for 21 days. Data represent means ± SEM, *n* = 10 rats per group

*P < 0.05, **P < 0.01, ***P < 0.001 vs. stressed saline-treated, saline-conditioned rats; #*P* < 0.05, ##*P* < 0.01, ###*P* < 0.001 vs. stressed imipramine-treated, saline-conditioned rats; ^*P* < 0.05, ^^*P* < 0.01, ^^^*P* < 0.001 vs. stressed saline-treated, nicotine-conditioned rats (Tukey’s test)

A post hoc analysis showed that imipramine administered to the 3 days nicotine-conditioned rats subjected to the CUMS procedure decreased the values of TAC in the cortex (*P* < 0.05), decreased the values of GPx in the brain (*P* < 0.001) and hippocampus (*P* < 0.01), decreased the values of SOD in the brain (*P* < 0.01) and cortex (*P* < 0.001), increased the values of MDA in the brain, cortex, hippocampus, and cerebellum (*P* < 0.001) as compared with imipramine-treated, saline-conditioned animals. Moreover, imipramine administered to the saline-conditioned rats subjected to the CUMS procedure increased the values of GPx in hippocampus (*P* < 0.05), as compared with stressed, saline-treated, saline-conditioned animals. Imipramine administered to stressed nicotine-conditioned rats also increased the values of TAC in cortex and MDA in the brain (*P* < 0.05) as compared with saline-treated, nicotine-conditioned animals.

## Discussion

Addiction is a complex disorder, as many factors contribute to the development and maintenance of this psychological and neurological disorder. Stress is also one of the key factors in facilitating reward associated with initial and prolonged drug exposure [4]. Stress produces a cascade of physiological and psychological effects, each with a distinctive time course. Thus, the relationship between stress and effects of drug of abuse, specifically those of nicotine, is not fully coherent and understood.

In the first series of experiments, we aimed to evaluate the relationship between nicotine administration and the CUMS in rats using the CPP paradigm widely used to study the rewarding effects of psychoactive substances. The CUMS model presents good validity and has been broadly used to investigate some of the physiological and behavioral consequences of chronic stress [32, 64, 66, 67]. Thus, in the present study, animals were either unstressed or exposed for 3 weeks to the CUMS procedure and subsequently tested in the CPP model. In this set of the study, we demonstrated that chronic mild stress may exacerbate nicotine-induced CPP as we found that, compared with nonstressed rats, animals submitted to the CUMS procedure had an increased response to nicotine-rewarding properties in the CPP test. Thus, this is the first study that demonstrates a facilitative role of stress in the initial rewarding effects of nicotine. Specifically, our results show that nicotine after 3 days of conditioning induced a clear-cut CPP. This finding agrees with earlier observations showing that nicotine induces CPP in adolescent and adult rats [54, 55, 68–72], also depending on strain used [73]. After 2 days of conditioning, nicotine provoked the CPP only in stressed rats. It is possible that stress serves to enhance nicotine’s initial rewarding effects which could have lasting implications for the development of dependence. The rewarding effects of nicotine in stressed rats were abolished by metyrapone, a glucocorticosteroid receptor antagonist. As for imipramine, this antidepressant diminished the rewarding effects of nicotine in stressed rats (after 2 days of conditioning) without any statistically significant effects in non-stressed rats (after 3 days of conditioning) supporting the effects of anhedonia-related behavior induced by the CUMS procedure in the acquisition of reward-related effects of nicotine.

It has been already supported that an acute stress enhances the rewarding effects of several addictive drugs (i.e. psychostimulants and opioids) in rodents as measured using the CPP paradigm [2, 29, 74–76] and exposure to a mild stressor produced an increase in ethanol consumption [77]. This finding supports clinical demonstrations that traumatic and stressful experiences can be associated with enhanced risk of drug abuse disorders.

In the context of the present study, it should be added that, in the search for psychological sources of addiction, particular attention is paid to the role of stress and some common mechanisms of both phenomena as the exact mechanisms by which this effect occurs have not been fully elucidated. Recent evidence points to stressor-induced potentiation of dopamine release within the mesocorticolimbic pathway [78], a critical mediator of the rewarding effects of nicotine and other abused drugs [79–81]. Moreover, a variety of acute stressors increase extracellular dopamine level in the nucleus accumbens [78, 82], providing further evidence for stressor-induced potentiation of dopaminergic transmission. Some of these effects have been shown to last for at least 24 h [83, 84], suggesting that an acute stress may enhance drug (e.g. nicotine) reward via a long-lasting potentiation. This fact is also important providing that the mescorticolimbic dopaminergic pathway is thought to be the critical neurobiological substrate for nicotine CPP [80, 81]. There is growing evidence that stress induces long-lasting adaptations that serve to potentiate activity within this pathway in a manner that may underlie the enhancing effects of stress on nicotine reinforcement. For instance, an acute restraint stress has been shown to increase long-lasting burst firing in putative midbrain dopaminergic neurons [85]. Accordingly, it has been demonstrated that the acute forced swim stress enhances strength at excitatory synapses and decreases strength at inhibitory synapses onto midbrain dopamine neurons [83, 84]. It is possible that prior exposure to an acute stressor facilitates nicotine CPP acquisition, as shown in our study, via induction of lasting synaptic changes in critical dopaminergic reward pathways.

It is also worth mentioning that stress activates the sympatho-adrenomedullar system and the HPA axis [86]. Activation of the HPA axis subsequently stimulates the secretion of adrenal glucocorticoids into the bloodstream. Thus, corticosterone acts as one of the biological factor mediating reward as more recent studies confirm the interaction between glucocorticoids and dopaminergic systems. In fact, it has been shown that an increase in the plasma level of glucocorticoids can cause the increase in dopamine level, also in the nucleus accumbens, and release of corticosterone following stressor exposure may thus also contribute to stress-induced enhancement of drug reward [45]. Previous studies have shown that corticosterone has positive reinforcing effects; this finding has also demonstrated that increase in corticosterone levels during stress has no aversive effects, but it is rewarding as it has positive reinforcing effects and is self-administered by rats [87, 88]. Glucocorticoids have been shown to increase dopaminergic response to psychostimulants and opioid drugs that are observed in stressed subject. On the other hand, the effects of corticosterone are dose- and glutamatergic receptor-dependent, with a low dose of corticosterone potentiating the NMDA-induced increase of dopamine, and high dose enhancing the AMPA-induced increase in GABA level [89]. The role of glucocorticosteroids and the HPA axis in the dependence-related behavior has also been confirmed in our study.

It should be noted that the present results could also be explained by non-reward mechanisms, such as the effects of stress on learning. CPP is a learning task based on classical conditioning as mentioned above, though the relationship between stress and learning in rodents is complex [50]. Acute stressor exposure has been shown to enhance learning in Pavlovian tasks, particularly when there is a delay between the stressor and onset of training. Accordingly, acute restraint enhances learning of subsequent context-dependent fear conditioning in rodents [90]. It is possible that the CUMS facilitated acquisition of CPP in the present study due to a general enhancement of learning by stress. As already stated, stress changes the dopamine levels in the brain regions receiving dense inputs from the ventral tegmental area, like the nucleus accumbens and basolateral amygdala [88, 91]. On the other hand, the hippocampus and the amygdala play inhibitory and excitatory roles, respectively, on the HPA axis activity [89]. Recent evidence implicate the amygdala in learning process related to rewards and punishments, and can also mediate craving and motivational significance to drug-associated cues and contexts [91]. As such, stress hormones released can then modulate memory strength via the amygdala, which in turn acts on sites of memory storage such as the prefrontal cortex. Several lines of evidence have implicated the amygdala as a substrate for stress-related modulation of memory and hippocampal functions also providing its role in the CPP acquisition and retention [92, 93]. Considering the previous investigations and our obtained data, it can be concluded that the effects of stress on reward pathway and its interactions with memory-related mechanism can be modulated via HPA activation. However, more studies focusing on the molecular pathways and physiological properties of the neurons during the simultaneous exposure to stress and drugs of abuse are necessary to have a better insight about the interaction between these crucial neuronal mechanisms. Thus, it is possible that prior CUMS procedure enhanced nicotine CPP acquisition due to nicotine’s ability to counteract stress-induced affective disorders like anxiety and depression (i.e. anhedonia) or even by facilitating nicotine’s conditioned anxiolytic and antidepressant effects, which along with reward may underlie nicotine’s addictive properties. From our study, the inhibitory effect of an antidepressant imipramine on nicotine CPP in stressed rats can further confirm this hypothesis.

Concerning our biochemical analysis, a growing body of evidence has suggested that repeated and unpredictable stress situations can have strong impact on production of ROS in the brain. And, the brain structures, which are participating in stress response and therefore are mainly affected by chronic exposure to stress, are prefrontal cortex and hippocampus, the structures involved in emotional and cognitive processes. In this context, ROS may play main role in the pathogenesis and development of neurological as well as psychiatric diseases like depression or bipolar disorder [94, 95]. Our study reported significant decrease in total antioxidant capacity (TAC), increase in activity of GPx, one of the antioxidant enzymes as well as significant increase in MDA concentration, a main marker of lipids peroxidation in all examined brain structures, which has been also described in our previous study [58].

Nicotine has been believed to have anti-stress effect as people in stress often smoke more as they feel more relaxed then [58]. Unfortunately, it is only a psychological impression, as nicotine activates the HPA axis on the stage of ACTH excretion and therefore increases the level of cortisol liberated to blood stream [96]. However, numerous studies have proved pro-oxidative properties of nicotine especially within the liver, kidneys, heart, and brain of experimental animals in vivo [97]. In our previous study, acute and subchronic nicotine treatment induces oxidative stress within brain structures responsible for cognitive processes [8, 58]. These studies as well as the results of present experiments showed significant decrease in total antioxidant status/capacity (TAS/TAC), increase in concentration of MDA, and suppression of antioxidant enzymes (SOD and GPx) activities after nicotine administration, what was even more significant in the case of stressed animals. These data strongly confirmed pro-oxidative effect of nicotine administered at single and repeated doses and proved that nicotine potentiates stress-induced oxidative stress on the level of lipids peroxidation, total antioxidant status, and antioxidant enzymes activity.

As disturbances in oxidant-antioxidant status may play important role in cortisol-induced depressive illnesses, therefore, it seems reasonable to check if antidepressant treatment would reverse the effects of oxidative stress. Therefore, we investigated the effect of imipramine on the markers of antioxidant barrier damage in the brain and its structures in the CUMS- and/or nicotine-conditioned animals. Unfortunately, our study did not reveal any antioxidant effects of imipramine against the CUMS nor nicotine administration. However, numerous studies have suggested positive impact of imipramine on oxidative changes caused by the CUMS [98, 99]. Imipramine treatment has been found to reverse lipid peroxidation in rat brain [100] as well as reversed other biochemical alterations in stressed animals like MDA level, nitrite, glutathione, and catalase activity [101]. The differences in the results of the experiments may come from duration of imipramine treatment. We have investigated only acute imipramine administration, while positive results have been obtained at subchronic or chronic administration.

Similarly, we aimed to estimate the impact of metyrapone, a corticosterone synthesis inhibitor, on chosen parameters of antioxidant barrier. Our study revealed that even single dose of metyrapone was able to counteract CUMS-induced oxidative stress especially at the level of lipids peroxidation process. Other studies have also reported that metyrapone administered from third to seventh day of rats’ exposure to hypobaric hypoxia [102] decreased ROS production and lipid peroxidation and increased oxidized and reduced glutathione levels in comparison to saline-treated control group. The authors have found significant improvement of antioxidant barrier parameters, which were correlated with decrease in corticosterone concentration in animals’ blood samples. The positive effect of metyrapone in our experiment was even better noticed in the case of nicotine-conditioned stressed animals, which may prove that metyrapone does not lower the overall cortisol level, but it prevents its increased nicotine-induced release to blood stream [96]. Moreover, like in the case of imipramine, the positive effects are more significant in the case of chronic or subchronic treatment with metyrapone.

In conclusion, the present study was aimed at investigating the nicotine reward-related action in rats exposed to the chronic stress model. To this purpose, rats were subjected to 3 weeks of chronic unpredictable stressful stimuli, after which the animals were submitted to the CPP paradigm. It has been shown that CUMS-exposed animals exhibited a clear CPP after only 2 days of conditioning with nicotine. Taking into consideration that oxidative stress is probably one of the key factors in the pathophysiology of depression, antioxidant effect of antidepressants as well as pharmacological anti-stress therapy could have beneficial results in prevention and/or treatment of that disease. Thus, detailed biochemical and neurochemical studies are required to reveal exact mechanism of oxidative theory of depression.

Understanding the mechanisms by which stress regulates the rewarding properties of drugs of abuse provides valuable insight into potential treatments for drug abuse. Therefore, determining the influence of the CUMS on addictive behaviors is a crucial, yet challenging, and complex task. Additional investigations are needed to fully characterize the effects of chronic stress on nicotine reward-related behaviors.
